# Predictors of Adverse Perinatal Outcome in Twin Anemia Polycythemia Sequence: Evidence From a Single Center Cohort Study

**DOI:** 10.1002/pd.70003

**Published:** 2025-10-20

**Authors:** Keren Zloto, Noa Rosenthal, Stav Cohen, Hagai Avnet, Boaz Weisz, Yoav Yinon

**Affiliations:** ^1^ Department of Obstetrics and Gynecology Fetal Medicine Unit Sheba Medical Center Ramat Gan Israel; ^2^ Gray Faculty of Medical and Health Sciences Tel‐Aviv University Tel‐Aviv Israel

## Abstract

**Objective:**

To evaluate the perinatal outcome of monochorionic pregnancies complicated by Twin Anemia‐Polycythemia Sequence (TAPS) and identify prognostic factors associated with adverse outcomes.

**Methods:**

A retrospective study (2012–2024) analyzed TAPS cases at a tertiary center. Demographic, obstetric, and neonatal outcomes were compared between conservative management and interventions (intrauterine transfusion, laser surgery, or selective feticide). TAPS characteristics were also analyzed in three subgroups: deliveries before/after 32 weeks, CNS findings on MRI, and dual versus single/no survival.

**Result:**

Of 32 TAPS cases, 68.75% were spontaneous, and 31.25% followed TTTS laser treatment. Conservative management was used in 62.5%, while 21.9% received intrauterine transfusions, 9.4% underwent laser treatment, and 9.4% had selective feticide. Gestational age at diagnosis and delivery was earlier in the intervention group (25 vs. 27 weeks, *p* = 0.05; 30.6 vs. 32.6 weeks, *p* = 0.04). CNS abnormalities (25%) were linked to earlier diagnosis. Perinatal survival was 86%, with dual, single, and no survival rates of 78.1%, 15.6%, and 6.2%, respectively. Patients with single/no survival were characterized by an earlier gestational age at diagnosis of TAPS compared to those with dual survival (21 vs. 27 weeks, *p* < 0.01).

**Conclusion:**

Earlier TAPS diagnosis is associated with poorer outcomes, including reduced survival and increased risk of brain injury.

## Summary

- What is already known about this topic?
  ◦ TAPS is a chronic complication in monochorionic twins involving unequal blood flow through tiny placental vessels, causing large hemoglobin differences and risks such as brain injury, neonatal death, and neurodevelopmental impairment, particularly in anemic twins.
- What does this study add?
  ◦ This study suggests that earlier gestational diagnosis of TAPS is linked to more severe disease, lower survival, and higher risk of fetal brain injury.

## 1 Introduction

Twin anemia polycythemia sequence (TAPS), is a chronic form of unbalanced feto‐fetal transfusion through minuscule placental anastomoses in monochorionic twin pregnancies, resulting in a significant hemoglobin difference between the twins [1, 2]. The donor twin develops anemia, while the recipient twin experiences polycythemia.

TAPS can occur spontaneously or after a laser treatment for TTTS [3]. The spontaneous occurrence of TAPS affects 3%–5% of monochorionic twin pregnancies, while TAPS developing after laser treatment for TTTS has a reported incidence of 2%–16% [4, 5], which can be reduced by using the Solomon technique [4]. Antenatal diagnosis is based on the detection of increased peak systolic velocity (PSV) in the donor twin's MCA suggesting fetal anemia, and decreased or normal MCA velocities in the recipient twin indicating polycythemia, all without the presence of twin oligohydramnios‐polyhydramnios sequence [6, 7, 8].

TAPS is a relatively recently recognized condition, first described in 2006 [9]. Although the understanding of the pathogenesis of this condition has grown considerably, there is still no consensus on the optimal antenatal management approach for TAPS [10]. Treatment options currently include expectant management, preterm delivery, intrauterine transfusion (IUT), fetoscopic laser surgery and selective feticide [5].

The neonatal outcome may range from isolated hemoglobin differences to severe cerebral injury and neonatal death. Furthermore, TAPS is also associated with neurodevelopment impairment with a four‐fold higher odds for the surviving anemic twins to be affected compared with their polycythemic co‐twins [3].

Currently, the data in the literature regarding prognostic factors for TAPS, which can assist in determining the optimal antenatal management, is limited. The aim of this study was to identify prognostic factors in monochorionic twin pregnancies complicated by TAPS which are associated with adverse perinatal outcomes.

## 2 Material and Methods

This was a retrospective cohort study of all patients with monochorionic twin pregnancies diagnosed with TAPS at a single tertiary center between 2012 and 2024. An electronic medical record chart review was conducted to identify all monochorionic twin pregnancies with a diagnosis of TAPS either spontaneously or post‐laser.

All monochorionic twin pregnancies referred to our center underwent a comprehensive evaluation every 2 weeks from 16 weeks of gestation, which included standard biometry, assessment of amniotic fluid volume in each sac, and Doppler flow measurements of the umbilical artery, middle cerebral artery peak systolic velocity (MCA‐PSV), and ductus venosus. After fetoscopic laser treatment for TTTS or when TAPS was suspected, a neurosonogram and fetal brain MRI were conducted between 28 and 32 weeks of gestation.

The antenatal diagnosis of TAPS was based on discordant MCA‐PSV measurements, with the TAPS donor having an MCA‐PSV greater than 1.5 Multiples of the Median (MoM), indicating fetal anemia, and the TAPS recipient showing an MCA‐PSV below 1 MoM, suggesting fetal polycythemia, without signs of TTTS. Additionally, an MCA‐PSV difference greater than 0.5 Mom between the twins, in the absence of TTTS, was also considered diagnostic [11]. Staging was performed according to the 2010 Slaghekke et al. classification [12] or the 2019 Tollenaara et al. [13]. classification. Postnatal diagnosis was confirmed by inter‐twin hemoglobin difference of more than 8 g/dL and with at least one of the following: a reticulocyte count ratio of > 1.7 or the presence of only minuscule vascular anastomoses (diameter < 1 mm) [14]. The decision to intervene or to follow conservatively was based on gestational age at diagnosis, severity, and patients' desire. In cases detected prior to 26 weeks, laser ablation was considered when there were signs of deterioration of either twin (worsening of MCAPSV of the anemic fetus or appearance of hydrops), IUT was considered in cases detected between 26 and 32 weeks with signs worsening fetal anemia and after 32 weeks early delivery was the treatment of choice.

Data for this study were extracted from a comprehensive computerized perinatal database. Maternal records were reviewed to assess demographic variables and TAPS characteristics, including gestational age at diagnosis, TAPS stage, spontaneous TAPS versus post‐laser TAPS, conservative management versus active management (IUT of the anemic fetus without partial exchange transfusion for the polycythemic fetus, fetoscopic laser surgery, and selective feticide), additional ultrasound findings (starry sky liver in the polycythemic twin, hydrops in the anemic twin and placental color discrepancy), and abnormal CNS findings on ultrasound and MRI. Obstetric characteristics, such as chorioamnionitis, preterm premature rupture of membranes (PPROM), hypertensive disorders of pregnancy (HDP), gestational diabetes mellitus (GDM), placental abruption, intrauterine fetal demise, mode of delivery, and preterm birth (PTB), were collected. Neonatal records were assessed for key outcomes, including birth weight, Apgar scores, admission to the neonatal intensive care unit, respiratory distress syndrome, intraventricular hemorrhage, necrotizing enterocolitis, need for mechanical ventilation, hyperbilirubinemia requiring treatment, neonatal hypoglycemia (defined as capillary blood glucose < 40 mg/dL or the need for oral/IV glucose), culture‐proven sepsis, postnatal hemoglobin, and neonatal death.

Demographic data, TAPS characteristics, as well as obstetric, labor, and neonatal outcomes were compared between the active management group (IUT, fetoscopic laser surgery, and selective feticide) and the conservative management group. Furthermore, TAPS characteristics were analyzed across three subgroups: deliveries before versus after 32 weeks of gestation, presence versus absence of abnormal fetal CNS findings, and dual survival versus single or no survival.

The study was approved by the ethics committee of Sheba Medical Center and informed consent was waived because of the retrospective nature of the study (1552‐24‐SMC).

Continuous variables were analyzed using the non‐parametric Mann–Whitney test, while the Chi‐Squared and Fisher's Exact tests were used for comparison between categorical variables.

Multivariate logistic regression analysis was conducted to identify factors associated with (1) delivery before versus after 32 weeks of gestation, (2) presence versus absence of abnormal fetal CNS findings, and (3) dual versus single or no fetal survival, while adjusting for potential confounders. The model included gestational age at diagnosis, management approach (intervention vs. conservative), and TAPS type (post‐laser vs. spontaneous) as covariates.

All statistical tests were two‐tailed, and a *p*‐value of less than 0.05 was considered statistically significant. All calculations were performed using IBM SPSS Statistics, version 25.

## 3 Results

During the study period, 1544 monochorionic diamniotic twin pregnancies were assessed at our center. Of these, 39 cases were diagnosed prenatally with TAPS (2.46%), of which seven did not meet the postnatal criteria for TAPS, resulting in 0.32 pregnancies involving 57 neonates who were included.

The demographic and TAPS characteristics are summarized in Table 1. The median gestational age at diagnosis was 25 weeks. Among the cases, 22 (68.75%) were spontaneous TAPS, whereas 10 (31.25%) were diagnosed following laser treatment for TTTS. A total of 20 patients (62.5%) were managed conservatively, 7 (21.9%) received intrauterine transfusions (IUT), 3 (9.4%) underwent laser treatment, and 3 (9.4%) had selective reduction. Spontaneous resolution occurred in 3 cases (9.4%), including two cases of TAPS stage 1 and one case of stage 2, diagnosed at 27, 32 and 23 weeks of gestation. Notably, none of these cases occurred following laser treatment. Abnormal CNS findings on ultrasound or MRI were observed in 8 cases (25% of pregnancies, 15.8% of fetuses), and included a small vermis (below the third percentile) with hypoplasia of its inferior part, a cerebellum measuring in the fifth percentile, asymmetry of the ventricles, and hemorrhages affecting the cerebellar lobes and vermis. The perinatal survival rate was 86% (55/64) with dual, single and no survival rates of 78.1% (25/32), 15.6% (5/32), and 6.2% (2/32), respectively.

**TABLE 1:** All cohort characteristics.

	All cohort *n* = 32
Maternal age	31 (28–34)
Gravidity	2.5 (1–4)
Parity	1 (0–2)
IVF treatment	6 (18.75)
Gestational age at diagnosis (weeks)	25 (23.25–29.75)
TAPS stage	
Stage 1	8 (25)
Stage 2	22 (68.75)
Stage 3	0 (0)
Stage 4	2 (6.25)
Stage 5	0 (0)
TAPS post laser	10 (31.25)
TAPS management	
Conservative management	20 (62.5)
Any intervention	12 (37.5)
IUT	7 (21.9)
Laser treatment	3 (9.4)
Selective reduction	3 (9.4)
SIUGR	6 (18.75)
Interval between laser and TAPS (days)	18 (3.75–33)
MCA‐PSV in MOM‐anemic twin at diagnosis	1.56 (1.38–1.9)
MCA‐PSV in MOM‐polycythemic twin at diagnosis	0.75 (0.68–0.8)
Delta MCA PSV in MOM at diagnosis	0.82 (0.57–1.19)
MCA‐PSV in MOM‐anemic twin at last follow‐up	1.49 (1.2–1.8)
MCA‐PSV in MOM‐polycythemic twin at last follow‐up	0.71 (0.57–0.89)
Delta MCA PSV in MOM at last follow‐up	0.76 (0.35–1.16)
Placenta color discrepancy	4 (12.5)
Stary sky liver for polycythemic	15 (46.9)
Hydrops for anemic	2 (6.25)
Spontaneous resolution	3 (9.4)
Abnormal CNS findings on US or MRI	8 (25)
IUFD^a^	4 (12.5)
Number of livebirths	
None	2 (6.25)
Single	5 (15.6)
Dual	25 (78.1)

*Note:* Data are presented as *n* (%) or median (interquartile range).

^a^ Calculated based on all fetuses, *N* = 76.

A comparison of demographic, TAPS, and neonatal characteristics between conservative and active management is presented in Tables 2, 3, 4. A total of 12 patients (37.5%) were included in the intervention group. Notably, one patient underwent IUT, laser treatment, and eventually selective reduction. The rate of post‐laser TAPS was higher among the intervention group (66.7% vs. 10%, *p* < 0.01) and the gestational age at diagnosis was earlier in the intervention group (25 vs. 27 weeks, *p* = 0.05). The gestational age at delivery was significantly earlier in the intervention group (30.6 vs. 32.6 weeks, *p* = 0.04), and accordingly the rate of preterm labor before 32 weeks of gestation was higher in this group (50% vs. 35%, *p* = 0.02). Neonatal data were comparable between the groups, except for birth weight, which was lower in the intervention group, and the rate of hyperbilirubinemia, which was higher (1340 vs. 1685 g, *p* < 0.01; 50% vs. 12%, *p* = 0.03).

**TABLE 2:** Demographic and obstetric characteristics.

	Intervention *n* = 12	Control *n* = 20	*p* value
Age (years)	32.5 (28–34.75)	30.5 (28–33.75)	0.41
Gravidity	2 (1.25–3.75)	3 (1–4)	0.57
Parity	1 (0.25–2)	1 (0–2)	0.73
Body mass index before pregnancy (kg/m^2^)	25.8 (19.6–28.65)	23.4 (20.4–26.8)	0.56
In vitro fertilization	3 (25)	3 (15)	0.64
PPROM	1 (8.3)	2 (10)	1
Hypertensive disorder of pregnancy	2 (16.7)	1 (5)	1
GDM	1 (8.3)	4 (20)	0.62
Placental abruption	1 (8.3)	0 (0)	0.38
Chorioamnionitis	1 (8.3)	1 (5)	1
Gestational age at delivery (weeks)	30.6 (28.6–33.1)	32.6 (31.6–34.6)	**0.04**
Cesarean delivery	9 (75)	14 (70)	0.76
Elective	6 (50)	7 (35)	0.55
NRFHR	2 (16.7)	3 (15)	1
Preterm labor < 24	N/A	N/A	N/A
Preterm labor < 28	1 (8.3)	1 (5)	1
Preterm labor < 32	9 (50)	7 (35)	**0.02**
Preterm labor < 34	10 (83.3)	12 (60)	0.22

*Note:* Data are presented as *n* (%) or median (interquartile range). Bolded if significantly different.

Abbreviations: GDM, gestational diabetes mellitus; IUFD, intrauterine fetal demise; NRFHR, non‐reassuring fetal heart rate; PPROM, preterm (< 37.0 weeks) premature rupture of membranes.

**TABLE 3:** TAPS characteristics.

	Intervention *n* = 12	Control *n* = 20	*p* value
Gestational age at diagnosis (weeks)	25 (19.5–25)	27 (24.25–30)	**0.05**
TAPS stage			
Stage 1	2 (16.7)	6 (30)	0.18
Stage 2	8 (66.7)	14 (70)	
Stage 3	0 (0)	0 (0)	
Stage 4	2 (16.7)	0 (0)	
Stage 5	0 (0)	0 (0)	
Post laser TAPS	8 (66.7)	2 (10)	**<** **0.01**
IUT	7 (58.3)	0 (0)	**<** **0.01**
Laser management	3 (25)	0 (0)	**0.04**
Selective reduction	3 (25)	0 (0)	**0.04**
SIUGR	4 (33.3)	6 (30)	0.16
MCA‐PSV in MOM‐anemic twin at diagnosis	1.7 (1.37–2.03)	1.51 (1.38–1.71)	0.14
MCA‐PSV in MOM‐polycythemic twin at diagnosis	0.78 (0.71–0.8)	0.87 (0.64–0.84)	0.25
Delta MCA PSV in MOM	0.98 (0.59–1.26)	0.74 (0.54–1.06)	0.17
MCA‐PSV in MOM‐anemic twin at last follow‐up	1.62 (1.24–1.91)	1.48 (1.15–1.7)	0.33
MCA‐PSV in MOM‐polycythemic twin at last follow‐up	0.65 (0.51–0.8)	0.75 (0.62–0.9)	0.33
Delta MCA PSV in MOM	1.09 (0.37–1.3)	0.75 (0.32–0.92)	0.29
Placenta color discrepancy	1 (8.3)	3 (15)	1
Stary sky liver for polycythemic	7 (58.3)	8 (40)	0.31
Hydrops for anemic	1 (8.3)	1 (5)	1
Spontaneous resolution	0 (0)	3 (15)	0.27
Abnormal CNS findings on US or MRI	3 (25)	5 (25)	1
IUFD	1 (8.3)	3 (15)	1
Number of livebirths			
None	1 (8.3)	1 (5)	0.42
Single	3 (25)	2 (10)	
Dual	8 (66.7)	17 (85)	

*Note:* Data are presented as *n* (%) or median (interquartile range). Bolded if significantly different.

Abbreviations: CNS, central nervous system; IUFD, intrauterine fetal demise; IUT, intrauterine Transfusion; PPROM, preterm (< 37.0 weeks) premature rupture of membranes; SIUGR, selective intrauterine growth restriction; TAPS, twin anemia polycythemia sequence; TTTS, twin‐to‐twin transfusion syndrome.

**TABLE 4:** Neonatal data.

	Intervention *n* = 24	Control *n* = 40	*p* value
Gender (female)	12 (50)	22 (55)	0.39
Birthweight (gr)	1340 (1189–1547)	1685 (1505.5–1975)	**<** **0.01**
Birthweight percentile	41 (22.5–66.5)	41.5 (24.75–51)	0.9
Birthweight discordancy	13.33 (2.98–24.52)	14.8 (7.72–24.5)	0.89
Hb anemic twin (gr/dL)	8.43 (7.58–14.52)	10.71 (8.11–13.75)	0.66
Hb polycythemic twin (gr/dL)	22.49 (21.1–24.12)	23.38 (21.72–24.18)	0.54
Hb difference	14 (11.73–15.95)	13.7 (10.06–15.85)	0.5
Blood transfusion	9 (37.5)	12 (30)	0.53
PH vein	7.29 (7.24–7.3)	7.29 (7.22–7.34)	0.99
Apgar score < 7 at 5 min	1 (4.2)	1 (4.2)	1
NICU admission (days)	16 (66.7)	27 (67.5)	0.65
Hypoglycemia	7 (29.2)	15 (37.5)	0.33
Hyperbilirubinemia	12 (50)	12 (30)	**0.03**
RDS	11 (45.8)	15 (37.5)	0.45
IVH	2 (8.3)	3 (7.5)	1
Sepsis	5 (20.8)	5 (12.5)	0.42
Mechanical ventilation	6 (25)	11 (27.5)	0.81
NEC	0 (0)	1 (2.5)	1
Postnatal CNS findings on US	8 (33.3)	10 (25)	0.44
Post natal echo findings	2 (8.3)	5 (12.5)	0.69
Neonatal death	N/A	N/A	N/A
Congenital anomaly	4 (16.7)	1 (2.5)	0.05
Composite outcome^*^	14 (58.3)	20 (50)	0.51

*Note:* Data are presented as *n* (%) or median (interquartile range). Bolded if significantly different.

Abbreviations: IVH, intraventricular hemorrhage; LGA, large for gestational age; NEC, necrotizing enterocolitis; NICU, neonatal intensive care unit; RDS, Respiratory distress syndrome; SGA, Small for gestational age.

*Composite outcome‐RDS, IVH grade 3–4, NEC, mechanical ventilation and neonatal death.

Table 5 presents a comparison of TAPS characteristics according to gestation age at delivery, the presence or absence of abnormal fetal CNS findings, and dual survival versus single or no survival. Sixteen patients (50%) delivered before 32 weeks, with significantly higher rates of post‐laser TAPS and IUT intervention in this group (62.5% vs. 0%, *p* < 0.01; 43.75% vs. 0%, *p* < 0.01). Conversely, a higher rate of patients who delivered after 32 weeks were managed conservatively (81.25% vs. 43.75%, *p* = 0.02).

**TABLE 5:** TAPS characteristics according to pregnancy outcomes.

	Preterm < 32 *n* = 16	Delivery after 32 weeks of gestation *n* = 16	*p* value	CNS finding *n* = 8	No CNS finding *n* = 24	*p* value	Dual survival *n* = 25	Single/no survival *n* = 7	*p* value
Gestational age at diagnosis (weeks)	25.7 (23–27.7)	29 (24–32.2)	0.13	22 (19–26.75)	26 (25–30)	**0.05**	27 (25–30)	21 (19–25)	**<** **0.01**
TAPS stage									
Stage 1	4 (25)	4 (25)	0.59	2 (25)	6 (25)	0.8	6 (24)	2 (28.6)	0.47
Stage 2	10 (62.5)	12 (75)		5 (62.5)	17 (70.8)		18 (72)	4 (57.1)	
Stage 3	0 (0)	0		0 (0)	0 (0)		0 (0)	0 (0)	
Stage 4	2 (12.5)	0		1 (12.5)	1 (4.2)		1 (4)	1 (14.3)	
Stage 5	0 (0)	0		0 (0)	0 (0)		0 (0)	0 (0)	
Post laser TAPS	10 (62.5)	0	**<** **0.01**	3 (37.5)	7 (29.2)	0.66	6 (24)	4 (57.1)	0.16
Conservative management	7 (43.75)	13 (81.25)	**0.02**	5 (62.5)	15 (62.5)	1	17 (68)	3 (42.9)	0.37
Any intervention	9 (56.25)	3 (18.75)	**0.02**	3 (37.5)	9 (37.5)	1	8 (25)	4 (57.1)	0.37
IUT management	7 (43.75)	0 (0)	**<** **0.01**	1 (12.5)	6 (25)	0.64	6 (24)	1 (14.3)	0.58
Laser management	2 (12.5)	1 (6.25)	1	2 (25)	1 (4.2)	0.14	1 (4)	2 (28.6)	0.11
Selective reduction management	2 (12.5)	1 (6.25)	1	2 (25)	1 (4.2)	0.14	0 (0)	3 (42.9)	**<** **0.01**
SIUGR	2 (12.5)	4 (25)	0.65	2 (25)	4 (16.7)	0.62	4 (16)	2 (28.6)	0.59
MCA‐PSV in MOM‐anemic twin at diagnosis	1.55 (1.28–1.95)	1.57 (1.39–1.76)	0.72	1.57 (1.41–1.95)	1.56 (1.34–1.9)	0.69	1.52 (1.35–1.84)	1.76 (1.49–2.06)	0.22
MCA‐PSV in MOM‐polycythemic twin at diagnosis	0.74 (0.68–0.79)	0.76 (0.67–0.86)	0.7	0.75 (0.71–0.86)	0.75 (0.65–0.79)	0.4	0.77 (0.69–0.83)	0.71 (0.65–0.77)	0.26
Delta MCA PSV in MOM	0.85 (0.54–1.25)	0.81 (0.57–1.14)	0.88	0.79 (0.53–1.25)	0.82 (0.57–1.19)	0.81	0.78 (0.55–1.18)	1.01 (0.67–1.35)	0.23
MCA‐PSV in MOM‐anemic twin at last follow‐up	1.6 (1.23–1.86)	1.41 (1.16–1.67)	0.29	1.17 (1.01–1.64)	1.52 (1.26–1.81)	0.12	1.48 (1.23–1.8)	1.64 (0.88–1.89)	0.86
MCA‐PSV in MOM‐polycythemic twin at last follow‐up	0.66 (0.57–0.87)	0.78 (0.62–0.91)	0.29	0.72 (0.68–0.79)	0.7 (0.56–0.94)	0.88	0.69 (0.56–0.86)	0.8 (0.72–0.9)	0.22
Delta MCA PSV in MOM	1.03 (0.36–1.33)	0.72 (0.33–0.87)	0.19	0.38 (0.29–1.03)	0.79 (0.4–1.23)	0.25	0.78 (0.35–1.21)	0.76 (0.26–1.09)	0.66
Placenta color discrepancy	1 (6.25)	3 (18.75)	0.6	1 (12.5)	3 (12.5)	1	2 (8)	2 (28.6)	0.2
Stary sky liver for polycythemic	9 (56.25)	6 (37.5)	0.28	5 (62.5)	10 (41.7)	0.42	10 (40)	5 (71.4)	0.2
Hydrops For anemic	1 (6.25)	1 (6.25)	1	0 (0)	2 (8.3)	1	1 (4)	1 (14.3)	0.39
Spontaneous resolution	1 (6.25)	2 (12.5)	1	0 (0)	3 (12.5)	0.55	3 (12)	0 (0)	1
Abnormal CNS findings on US or MRI	5 (31.25)	3 (18.75)	0.68	N/A	N/A	N/A	4 (16)	4 (57.1)	**0.02**
IUFD	2 (12.5)	2 (12.5)	1	2 (25)	2 (8.3)	0.25	0 (0)	4 (57.1)	**<** **0.01**
Number of livebirths									
None	2 (12.5)	0 (0)	0.65	1 (12.5)	1 (4.2)	0.06	0 (0)	2 (28.6)	**<** **0.01**
Single	2 (12.5)	3 (18.75)		3 (37.5)	2 (8.3)		0 (0)	5 (71.4)	
Dual	12 (75)	13 (81.25)		4 (50)	21 (87.5)		25 (100)	0 (0)	

*Note:* Data are presented as *n* (%) or median (interquartile range). Bolded if significantly different.

Abbreviations: CNS, central nervous system; IUFD, intrauterine fetal demise; IUT, intrauterine transfusion; PPROM, preterm (< 37.0 weeks) premature rupture of membranes; SIUGR, selective intrauterine growth restriction; TAPS, twin anemia polycythemia sequence; TTTS, twin‐to‐twin transfusion syndrome.

In eight pregnancies (25%), nine fetuses (15%) exhibited abnormal CNS findings on ultrasound or MRI. Of these, 6 (18.75%) were polycythemic and 3 (9.4%) were anemic. There was no significant difference in the prevalence of CNS findings between polycythemic and anemic twins. Of note, TAPS was diagnosed earlier in the CNS findings group (22 vs. 26 weeks, *p* = 0.05). This association remained statistically significant after adjusting for management approach and TAPS type in multivariate analysis (Table 6).

**TABLE 6:** Regression analysis of factors associated with pregnancy outcomes.

Outcome	Preterm < 32 *n* = 16	Delivery after 32 weeks of gestation *n* = 16	OR	*p* value	CI 95%	CNS finding *n* = 8	No CNS finding *n* = 24	OR	*p* value	CI 95%	Dual survival *n* = 25	Single/no survival *n* = 7	OR	*p* value	CI 95%
Gestational age at diagnosis (weeks)	25.7 (23–27.7)	29 (24–32.2)	0.947	0.62	0.76,1.17	22 (19–26.75)	26 (25–30)	0.73	**0.02**	0.56,0.96	27 (25–30)	21 (19–25)	1.57	**0.01**	1.09,2.25
Post laser TAPS	10 (62.5)	0	44	0.99	N/A	3 (37.5)	7 (29.2)	1.95	0.58	0.17,21.85	6 (24)	4 (57.1)	0.15	0.2	0.01,2.75
Any intervention	9 (56.25)	3 (18.75)	0.76	0.83	0.05,9.97	3 (37.5)	9 (37.5)	0.27	0.27	0.01,3.28	8 (25)	4 (57.1)	3.24	0.43	0.16,62.82

*Note:* Bold values indicate significantly different.

Seven patients (21.9%) were included in the single twin survival (five patients, 71.4%) or no survival (two patients, 28.6%) group. Patients with single/no survival were characterized by an earlier gestational age at diagnosis of TAPS compared to those with dual survival (21 vs. 27 weeks, *p* < 0.01). This association remained statistically significant after adjusting for management approach and TAPS type in multivariate analysis (Table 6).

Table 7 presents a comparison of TAPS characteristics between cases of post‐laser and spontaneous TAPS. Intrauterine transfusion (IUT) and preterm delivery before 32 weeks of gestation were significantly more frequent in the post‐laser TAPS group compared to spontaneous TAPS group (60% vs. 4.55%, *p* < 0.01; 100% vs. 27.3%, *p* < 0.01). Additionally, there was a trend toward lower dual survival in the post‐laser group (60% vs. 86.4%, *p* = 0.07).

**TABLE 7:** TAPS characteristics—post‐laser versus spontaneous TAPS.

	Post‐laser TAPS *n* = 10	Spontaneous TAPSl *n* = 22	*p* value
Gestational age at diagnosis (weeks)	25 (20.5–25.5)	27 (24.5–30.25)	0.06
Preterm < 32	10 (100)	6 (27.3)	**<** **0.01**
TAPS stage			
Stage 1	2 (20)	6 (27.3)	0.09
Stage 2	6 (60)	16 (72.7)	
Stage 3	0 (0)	0 (0)	
Stage 4	2 (20)	0 (0)	
Stage 5	0 (0)	0 (0)	
IUT management	6 (60)	1 (4.55)	**<** **0.01**
Laser management	2 (20)	1 (4.55)	0.22
Selective reduction management	2 (20)	1 (4.55)	0.22
SIUGR	2 (20)	4 (18.2)	1
MCA‐PSV in MOM‐anemic twin at diagnosis	1.8 (1.4–2.1)	1.5 (1.4–1.7)	0.16
MCA‐PSV in MOM‐polycythemic twin at diagnosis	0.8 (0.7–0.8)	0.7 (0.6–0.8)	0.58
Delta MCA PSV in MOM	1.1 (0.6–1.3)	0.7 (0.6–1)	0.18
MCA‐PSV in MOM‐anemic twin at last follow‐up	1.6 (1.3–2.1)	1.4 (1.2–1.7)	0.18
MCA‐PSV in MOM‐polycythemic twin at last follow‐up	0.6 (0.5–0.8)	0.75 (0.6–0.9)	0.23
Delta MCA PSV in MOM	1.2 (0.4–1.5)	0.7 (0.3–1)	0.06
Placenta color discrepancy	1 (10)	3 (13.6)	0.77
Stary sky liver for polycythemic	6 (60)	9 (40.9)	0.31
Hydrops for anemic	1 (10)	1 (4.55)	0.53
Spontaneous resolution	0 (0)	3 (13.6)	0.53
Abnormal CNS findings on US or MRI	3 (30)	5 (22.7)	0.66
IUFD	2 (20)	2 (9.1)	0.57
Number of livebirths			
None	0 (0)	2 (9.1)	0.07
Single	3 (30)	2 (9.1)	
Dual	6 (60)	19 (86.4)	

*Note:* Bold values indicate significantly different.

## 4 Discussion

In this study, we explored the characteristics of TAPS in relation to three perinatal outcomes: preterm labor before 32 weeks of gestation, the presence of fetal CNS injury and survival. As expected, the preterm labor group was characterized by a higher rate of post‐laser TAPS and intervention. Interestingly, earlier diagnosis of TAPS was associated with increased risk of fetal CNS injury and decreased survival, suggesting that early presentation of TAPS is a poor prognostic factor.

We have also shown that intervention for TAPS, as opposed to conservative management, was associated with a higher risk for preterm birth, reflecting the fact that intervention is usually preserved for more severe cases.

A large retrospective study by Tollenaar et al., which examined outcomes in 370 cases of TAPS and compared different antenatal management strategies (expectant management, delivery within 7 days of diagnosis, IUT, laser surgery, and selective feticide). Pregnancy prolongation was best achieved with expectant management, laser surgery, or selective feticide [5, 15]. In their study, there was no difference in the gestational age at diagnosis across the management groups, though perinatal morbidity was significantly higher in cases treated with IUT, which were delivered earlier compared to other management strategies [5].

Prematurity plays a critical role in the short‐ and long‐term health of TAPS twins, making pregnancy prolongation crucial for improving outcomes [5, 16, 17].

The earlier diagnosis as well as the elevated rate of preterm birth among TAPS cases managed with intervention may primarily reflect disease severity, prompting the need for active treatment. Another possible interpretation is that earlier diagnosis increased the likelihood of intervention, which in turn led to higher rates of preterm delivery.

We have previously reported on the risk of fetal brain injury in pregnancies complicated by TAPS [1]. Prenatal brain lesions were detected in 13% of fetuses, correlating with long‐term studies, which found severe neurodevelopmental impairment in 9% of survivors [3, 16, 18]. The poor neurodevelopmental outcome related to TAPS emphasizes the need to identify factors associated with such outcomes. In our study, brain injury was detected in 15.8% of fetuses, and was more common in cases, which were detected earlier, suggesting that early presentation of TAPS reflects a more severe disease. This assumption is reinforced by our finding that earlier presentation of TAPS was also associated with decreased survival.

A recent large retrospective cohort study by Tollenaar et al., which included 249 TAPS cases, reported on high rates of adverse outcomes, including 15% neonatal mortality and 33% severe neonatal morbidity. Early gestational age at birth was identified as the strongest predictor of neonatal mortality and morbidity. The perinatal outcomes seem to be worse in post‐laser TAPS with a perinatal mortality rate of 25% and severe neonatal morbidity rate of 40% in one study [7]. In our cohort, 10 patients were diagnosed with post‐laser TAPS, all of whom delivered before 32 weeks of gestation and dual survival was lower in this group, nearly reaching statistical significance. It is noteworthy that in our cohort, among fetuses with abnormal CNS findings on ultrasound or MRI, 6 were polycythemic and 3 were anemic. In contrast, Tollenaar et al. reported severe neurodevelopmental impairment in 9% of spontaneous TAPS survivors, which was more frequently observed in the previous anemic compared with polycythemic twins (18%; 6/34 vs. 3%; 1/40). However, this difference did not reach statistical significance [19].

Spontaneous resolution of TAPS has been reported in several studies [5, 10]. Tollenaar et al. reported a 16% rate of spontaneous resolution among patients managed expectantly [5]. A case report by Couck et al. described a case of TAPS diagnosed at 15 weeks of gestation that was managed expectantly, with the MCA‐PSV of the donor improving at 19 weeks and normalizing by 21 weeks. After birth, the ex‐donor had a hemoglobin level of 28 g/dL and underwent partial exchange transfusion, whereas the ex‐recipient had a normal hemoglobin level. They hypothesized that as the placenta grew, the small anastomoses causing TAPS spontaneously closed between 15‐ and 19‐weeks of gestation [20].

In our study, spontaneous resolution was observed in three cases diagnosed at 23, 27 and 32 weeks of gestation. The underlying mechanism explaining this phenomenon is still unclear.

The strength of this study lies in its single‐center design, allowing for detailed and consistent documentation of pregnancy follow‐up and outcomes. Since TAPS was first introduced in the literature in 2006 [9], a considerable amount of data has been collected, but there remains uncertainty regarding the factors that influence clinical decisions with respect to active or expectant management [10].

Our study is not without limitations. The retrospective design introduces the potential for biases inherent to this type of research. Additionally, the cohort size is small and consists of both spontaneous and post‐laser TAPS patients, and consequentially may be underpowered to detect clinically important associations. Furthermore, the absence of data on long‐term outcomes restricts our ability to fully evaluate the impact of TAPS characteristics on neurodevelopmental outcomes.

In conclusion, our findings suggest that an earlier gestational age at diagnosis may be associated with a more severe form of TAPS, resulting in decreased survival and increased risk for fetal brain injury. These insights could assist clinicians in making more informed decisions regarding the appropriate management approach for each individual case. Further large‐scale studies are needed to validate this trend and clarify its clinical implications.

## Consent

The study was approved by the ethics committee of Sheba Medical Center and informed consent was waived due to the retrospective nature of the study (1552‐24‐SMC).

## Conflicts of Interest

The authors declare no conflicts of interest.

## Data Availability

Data available on request from the authors.
